# Supplementary material for: Microstructure Evolution, Constitutive Modelling, and Superplastic Forming of Experimental 6XXX-Type Alloys Processed with Different Thermomechanical Treatments
Source: Materials (Basel). 2023 Jan 3;16(1):445. doi: 10.3390/ma16010445 (PMC9821823; doi:10.3390/ma16010445)
Supplement: Supplementary file 1 [file materials-16-00445-s001.zip › materials-2113985-supplementary.pdf]

Supplementary Materials:

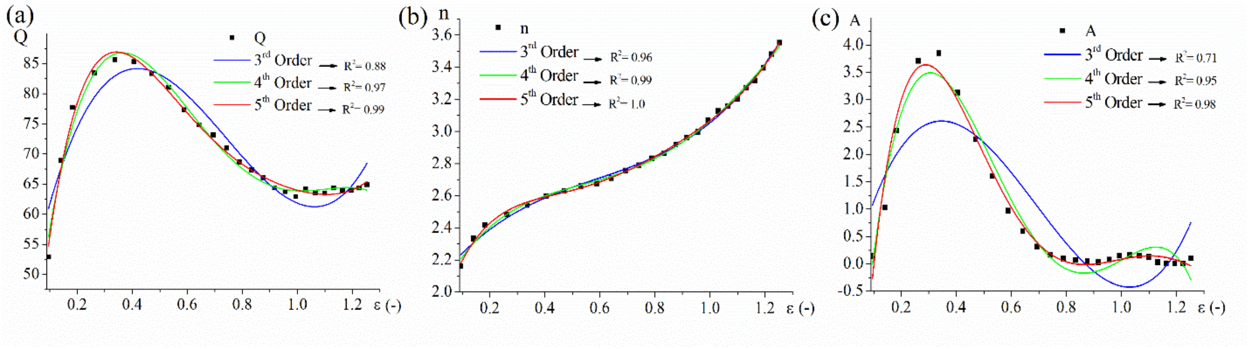

Figure S1. The material constants, Q, n, A, dependance on the strain of the investigated alloy at studied range of strain rate and temperature. It is noted that, in this work, the polynomial of degree 5 provides minimum level of errors, the higher order, as 6, insignificantly decreases the errors while increases the number of constants (correct to 1.0)

Table S1. Constants for Equation 8.

Parameter	Y0	B1	B2	B3	B4	B5	Adj. R-Square
Q	11.23	598.95	-1681.20	2072.93	-1222.08	284.17	0.99
n	1.74	6.10	-17.93	26.55	-18.37	4.98	1.0
A	-7.08	96.77	-301.30	389.33	-227.02	49.39	0.98

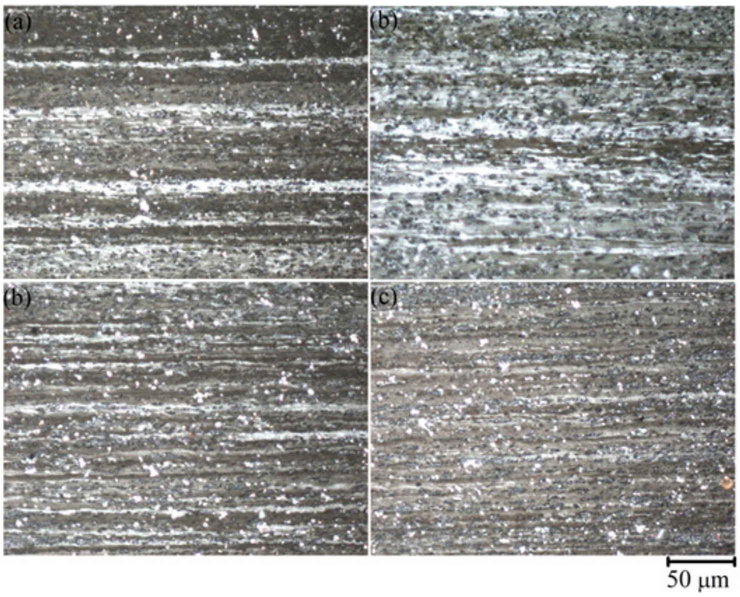

Figure S2. The grain structure of the mechanically treated samples in (a) 1HR, (b) 2HR, (c) CR50, and (d) CR80 regimes and annealed at 480 °C for 20 min (OM).
